# Therapeutic Impact of Human Serum Albumin–Thioredoxin Fusion Protein on Influenza Virus-Induced Lung Injury Mice

**DOI:** 10.3389/fimmu.2014.00561

**Published:** 2014-11-05

**Authors:** Ryota Tanaka, Yu Ishima, Yuki Enoki, Kazuhiko Kimachi, Tatsuya Shirai, Hiroshi Watanabe, Victor T. G. Chuang, Toru Maruyama, Masaki Otagiri

**Affiliations:** ^1^Department of Biopharmaceutics, Graduate School of Pharmaceutical Sciences, Kumamoto University, Kumamoto, Japan; ^2^Center for Clinical Pharmaceutical Sciences, School of Pharmacy, Kumamoto University, Kumamoto, Japan; ^3^The Chemo-Sero-Therapeutic Research Institute (KAKETSUKEN), Kumamoto, Japan; ^4^Curtin Health Innovation Research Institute, School of Pharmacy, Faculty of Health Sciences, Curtin University, Perth, WA, Australia; ^5^Faculty of Pharmaceutical Sciences, Sojo University, Kumamoto, Japan; ^6^DDS Research Institute, Sojo University, Kumamoto, Japan

**Keywords:** albumin, thioredoxin, fusion, anti-oxidization, influenza virus

## Abstract

Reactive oxygen species (ROS) are the primary pathogenic molecules produced in viral lung infections. We previously reported on the use of a recombinant human serum albumin (HSA)–thioredoxin 1 (Trx) fusion protein (HSA–Trx) for extending the half-life Trx, an endogenous protein with anti-oxidant properties. As a result, it was possible to overcome the unfavorable pharmacokinetic and short pharmacological properties of Trx. We hypothesized that HSA–Trx would attenuate the enhanced ROS production of species such as hydroxyl radicals by neutrophils during an influenza viral infection. The levels of 8-hydroxy-2′-deoxyguanosine and 3-nitrotyrosine were used as indices of the anti-oxidant activity of HSA–Trx. In addition, the cytoprotective effects of HSA–Trx were examined in PR8 (H1N1) influenza virus-induced lung injured mice. The findings show that HSA–Trx reduced the number of total cells, neutrophils, and total protein in BALF of influenza virus-induced lung injured mice. The HSA–Trx treatment significantly decreased the level of 8-hydroxy-2′-deoxyguanosine and 3-nitrotyrosine, but failed to inhibit inducible nitric oxide synthase expression, in the lungs of the virus-infected mice. On the other hand, Tamiflu^®^ treatment also significantly suppressed the production of inflammatory cells and neutrophil infiltration, as well as the protein level in BALF and lung histopathological alterations caused by the influenza virus. The suppressive effect of Tamiflu^®^ was slightly stronger than that of HSA–Trx. Interestingly, Tamiflu^®^ significantly decreased virus proliferation, while HSA–Trx had no effect. These results indicate that HSA–Trx may be of therapeutic value for the treatment of various acute inflammatory disorders such as influenza-virus-induced pneumonia, by inhibiting inflammatory-cell responses and suppressing the overproduction of NO in the lung.

## Introduction

Influenza virus infections cause a broad array of illnesses, including acute lung injuries (ALI), which are responsible for vital morbidity and mortality both in children and adults (1). Although most influenza A viruses cause a transitory localized debilitating respiratory illness in human beings, more severe respiratory and systemic complications that are sometimes fatal can arise, as evidenced by the pandemic influenza of 1918 caused by the H1N1 virus and the highly pathogenic avian H5N1 virus (2). In addition, the novel swine-origin influenza virus H1N1 that spread globally in 2009 was an attenuated strain, but children and patients with a pre-existing disease developed more severe infections (3).

There are several, currently available antiviral therapeutic agents, including neuraminidase inhibitors, oseltamivir (Tamiful^®^) and zanamivir (Relenza^®^) to combat seasonal and pandemic influenza A virus outbreaks (4). These antiviral agents that prevent the release of the influenza virus from already infected cells and the transmission of the virus are clearly effective in the treatment of such infections. However, emerging evidence suggests that certain strains of the influenza virus are developing resistance to these antivirals, and sometimes cause severe ALI, requiring intensive care and mechanical ventilation in clinical settings (5). Therefore, the development of newer, more effective therapeutic approaches that differ from traditional virus based strategies is of great importance.

Recent studies have suggested that oxidative stress plays an important role in the pathogenesis and development of influenza-induced ALI (6). At the time of an influenza infection, several cells of the innate immune system, such as alveolar macrophages and neutrophils release reactive oxygen species (ROS) and reactive nitrogen species (RNOS), including as superoxide $\left(O_{2}^{•-}\right)$, hydroxyl radical (^⋅^OH), nitric oxide (NO), and peroxynitrite (ONOO^−^) in order to combat the invading virus. However, the resulting overproduced ROS and RNOS not only exert a direct cytopathic effect on viral replication in the infected cells but also induced the apoptotic cell death of non-infected cells. That ROS and RNOS play critical roles as mediators of virus-induced lung damage is supported studies in which exogenous anti-oxidants, such as superoxide dismutase (SOD), catalase (anti-oxidative enzymes), allopurinol (xanthine oxidase inhibitor), or *N*-monomethyl-l-arginine (NO synthase inhibitor) decreased the extent of lung damage and mortality in influenza-infected mice (7–9). In addition, the overexpression of extracellular SOD or heme oxygenase-1 was reported to suppress lung injury and inflammation, resulting in an improved survival rate (10, 11).

Thioredoxin-1 (Trx) is a small redox-active protein (12 kDa) that is ubiquitously present in the human body and is one of the defense proteins that are induced in response to various oxidative stress conditions (12). In addition to its potent anti-oxidative effect, which is derived from dithiol-disulfide exchange in its active site, Trx also has anti-inflammatory properties, due mainly to its ability to inhibit neutrophil chemotaxis to inflammatory sites (13). Because of its desirable anti-oxidative and anti-inflammatory properties, Trx represents a new and potentially effective therapeutic agent for the treatment of influenza virus-induced ALI. However, since Trx is eliminated extensively via glomerular filtration, its plasma half-life is only about 1 h in mice and 2 h in rats, which is extremely short in terms of producing a significant therapeutic impact (13, 14). In order to obtain a satisfactory therapeutic outcome, a sustainable therapeutic concentration of Trx would be needed. To achieve this, a constant infusion or frequent repeated administrations of Trx would be required. In fact, Yashiro et al. reported that exogenous recombinant Trx was effective in inhibiting influenza virus-induced lung damage, when administered intraperitoneally 2 day intervals (15).

In an attempt to increase the blood retention time of Trx, we recently produced a genetically engineered fusion protein comprises human serum albumin (HSA) and Trx (HSA–Trx) using a *Pichia* expression system (16). The incentives for targeting albumin are that it constitutes the most abundant serum protein in blood, and albumin has an extraordinary long half-life of 2–3 weeks in human beings. In addition to having a molecular size above the renal clearance threshold, the long half-lives are attributed to the efficient receptor-mediated recycling pathway involving the neonatal Fc receptor (FcRn) (17). Actually, the plasma half-life of the HSA–Trx fusion protein in normal mice was found to be similar to that of HSA, which is 10 times longer than the plasma half-life of Trx itself. Interestingly, HSA–Trx showed a higher distribution in the lungs than Trx (16). Therefore, a further attempt will be made to investigate the clinical usefulness of HSA–Trx in treating oxidative stress and inflammation related lung disorders.

The purpose of this study was to investigate the therapeutic impact of HSA–Trx in the treatment of influenza-induced ALI. Using an influenza-induced ALI mouse model, the results showed that HSA–Trx could prevent this disease via its long acting anti-oxidative and anti-chemotaxis effects.

## Materials and Methods

### Experimental animals and reagents

Sea-ICR mice (5 weeks, male) were obtained from Kyudo Co., Ltd. (Saga, Japan). Blue Sepharose 6 Fast Flow, HiTrap Phenyl HP column and PD-10 desalting column were obtained from GE Healthcare (Tokyo, Japan). Chloral hydrate was obtained from Sigma (Tokyo, Japan). Diff-Quick reagents were purchased from Kokusai Shiyaku (Kobe, Japan). Coomassie Brilliant Blue solution and HistoVT One were obtained from Nacalai Tesque (Kyoto, Japan). Interferon-γ (IFN-γ) ELISA kit was purchased from Biolegend (San Diego, CA, USA). Mayer’s hematoxylin, a 1% eosin alcohol solution, mounting medium for histological examinations (malinol) were from Muto Pure Chemicals (Tokyo, Japan). 4′,6-Diamidino-2-phenylindole (DAPI) was obtained from Dojindo (Kumamoto, Japan). All other chemicals were of the highest analytical grades available.

### Production of HSA–Trx fusion protein

Human serum albumin, Trx, and the HSA–Trx fusion protein were produced following previously reported methods (18, 19). Transformed *Pichia pastoris* cells were incubated in 1250 ml of BMGY liquid media [1% yeast extract, 2% pepton, 100 mM potassium phosphate (pH 6.0), 1.34% yeast nitrogen base with ammonium sulfate without amino acids, 4 × 10^−5^% biotin, 1% glycerol] (growth phase) for 2 days (OD_600_ = 2), and was then cultured in 800 ml of BMMY media that contained a protein expression inducer as well as a carbon source, methanol [1% yeast extract, 2% pepton, 100 mM potassium phosphate (pH 6.0), 1.34% yeast nitrogen base with ammonium sulfate without amino acids, 4 × 10^−5^% biotin, 1% methanol] (protein induction phase) for 3 days at 30°C. Methanol was added at 12 h intervals to permit the concentration of methanol to be maintained at 1% in order to sustain the protein expression induction effect. Purification of the fusion protein was initially carried out by chromatography on a Blue Sepharose 6 Fast Flow column equilibrated with 200 mM sodium acetate buffer (pH 5.5) after dialysis against the same buffer. Using AKTA prime, a 5 ml HiTrap Phenyl HP column for hydrophobic chromatography, the following conditions were employed: Buffer A, 50 mM Tris-HCl/1.5 M ammonium sulfate, pH 7.0; Buffer B, 50 mM Tris-HCl, pH 7.0; Gradient, 0–100% (Buffer B) 100 ml; Flow rate, 3 ml/min. The fusion protein was analyzed by SDS-PAGE on a 10% polyacrylamide gel, with Coomassie blue R250 staining. The purity of the fusion protein was estimated to be in excess of 97%.

### Production of influenza-induced ALI mice model

Influenza virus A/Puerto Rico/8/34 (H1N1) was used throughout the experiments. All animal experiments were conducted in accordance with the guidelines of Center for Animal Resources and Development, Kumamoto University for the care and use of laboratory animals. All animal experiments were approved by the experimental animal ethics committee at the Kumamoto University (B 26-058). Influenza-induced ALI model mice were produced by intratracheal injection of influenza virus suspension diluted with LB medium at a dose of 1.5 × LD_50_ under anesthesia with chloral hydrate (500 mg/kg) on day 0. Either Trx or HSA–Trx was (but not both) administered intravenously (3.5 nmol protein in 200 μl saline/mouse) via the mice tail vein at 4 and 6 days after virus infection. Tamiful^®^ was administrated intraperitoneally (10 mg/kg/day, 2 times/day) for 4 days from 2 days after virus infection. The efficacy of therapeutic agents was evaluated with mice sacrificed at 8 days after virus infection.

### Analysis of lung lavage samples

The mice were anesthetized with diethyl ether, the chests were opened, and blood was sampled for measurement of hydroperoxides (described below). BALF was collected by cannulating the trachea and lavaging the lung with 1 ml of sterile PBS containing 50 U/ml heparin (two times). About 1.8 ml of BALF was routinely recovered from each animal. The BALF was centrifuged at 4,100 × *g* for 5 min at 4°C to separate the cells in the BALF from the liquid. Cells were suspended in 0.9% NaCl and the resulting lysate was centrifuged again. From the recovered cells, the total cell number was counted using a hemocytometer. Cells were stained with Diff-Quick reagents, and the ratios of alveolar macrophages, neutrophils, and lymphocytes to total cells were determined. More than 200 cells were counted for each sample. The protein concentration in BALF was measured with the Protein Assay Coomassie Brilliant Blue solution with BSA as a standard. IFN-γ levels in BALF were measured by ELISA, following the manufacturer’s suggested protocol.

### Histological examination of lung tissue

For the histological analysis, the recovered lung tissues were fixed in 10% neutral-buffered formalin (Wako, Osaka, Japan), embedded in paraffin (Sakura Finetek Japan, Tokyo, Japan) and sectioned at 4-μm thickness. The lung sections were subjected to hematoxylin and eosin (HE) staining for morphologic analysis, and immunohistochemistry for inducible nitric oxide synthase (iNOS) (N-20) (Santa Cruz Biotechnology, cat#: sc-651, Santa Cruz, CA, USA), 8-hydroxy-2′-deoxygenase (8-OHdG) (15A3) (Santa Cruz Biotechnology, cat#: sc-66036, Santa Cruz, CA, USA), or 3-nitrotyrosine (NO_2_-Tyr) (Millipore, cat#: AB5411, Billerica, MA, USA). For the immunohistochemistry of iNOS, 8-OHdG, or NO_2_-Tyr, antigen retrieval was first performed by means of HistoVT One. A solution containing 50 mM Tris-HCl (pH 7.4) and 0.1% Tween-20 (T-TB) was then used to solubilize the hepatic slices, followed by blocking with Block Ace (Dainippon Pharmaceutical, Osaka, Japan) at 25°C for 15 min. Next, reaction with the primary antibody was carried out overnight at a temperature below 4°C. In addition, the primary antibodies against iNOS, 8-OHdG, or NO_2_-Tyr were diluted (1:50) with 0.5% BSA in PBS before use and conjugated with DAPI solution (10 μg/ml). The hepatic slices were then washed with T-TB, followed by reaction with the secondary antibody at 25°C for 1.5 h. For iNOS and NO_2_-Tyr immunostaining, Alexa Fluor 488 goat anti-rabbit IgG (H + L) (Invitrogen, cat#: A-11034, Tokyo, Japan) and for 8-OHdG immunostaining, Alexa Fluor 555 goat anti-mouse IgG (H + L) (Invitrogen, cat#: A-21424, Tokyo, Japan) were diluted (1:200) with 0.5% BSA in PBS before use. After the reaction, the slide was observed under a microscope (Keyence, BZ-8000, Osaka, Japan). Image analyses of the extent and intensity of iNOS, 8-OHdG and NO_2_-Tyr immunostaining were also performed using the image J software.

### Measurement of plasma hydroperoxides

Plasma was prepared from recovered blood sample. The plasma concentration of hydroperoxides (whole oxidant capacity of plasma against *N*,*N*-diethylparaphenylene-diamine in acidic buffer) was measured using the FREE *carpe diem* (Diacron International, Grosseto, Italy) following the manufacturer’s recommended protocols. The measurement unit was CARR U. It has been established that 1 CARR U corresponds to 0.08 mg/dl hydrogen peroxide.

### Measurement of virus load in lung tissue

The left lungs from influenza virus-infected mice were removed, weighed, and homogenized in 1 ml of RPMI medium 1640. After centrifugation at 100 × *g* for 10 min at 4°C, the supernatant was recovered. Virus titers were determined by a plaque assay on Madin–Darby canine kidney (MDCK) cells. MDCK cells were seeded on a 24 well culture plate and cultured until reaching confluency. Cells were washed with PBS, and then 0.1-ml amounts of each dilution were inoculated to cultured cell followed by incubation for 1 h at 34°C. DMEM (0.5 ml) containing 100 U/ml penicillin, 100 μg/ml streptomycin, and 0.5 μg/ml fungizone was added to each well and incubated at 37°C for 3 days in a humidified incubator with 5% CO_2_. Cytopathological effect was calculated and 50% tissue culture infectious dose (TCID50) was calculated using the formula developed by Reed and Muench (20).

### Preparation of fluorescein isothiocianate-labeled HSA–Trx and evaluation of distribution to BALF of FITC-labeled HSA–Trx in influenza-infected mice

Human serum albumin–Trx was labeled with fluorescein isothiocianate (FITC). HSA–Trx (4 mg/ml) and FITC (1 mg/ml) were dissolved in 0.15 M K_2_HPO_4_ (pH 9.5), followed by mixing for 4 h at room temperature. The resulting solution was desalted by passage through a PD-10 desalting column and eluent was concentrated by Vivapore (Sartorius Stedim Biotech, A.S., France). FITC-labeled HSA–Trx (3.5 nmol/mouse) was administrated intravenously to normal mice or model mice at 4 or 6 days after virus infection. At 2 h after its administration, the mice were anesthetized with diethyl ether, the chests were opened, and blood was drained. BALF was collected by cannulating the trachea and lavaging the lung with 1 ml of sterile PBS containing 50 U/ml heparin. About 0.8 ml of BALF was routinely recovered from each animal. The BALF was centrifuged at 4,100 × *g* for 5 min at 4°C to separate the cells in the BALF from the liquid. The BALF levels of FITC-labeled HSA–Trx were quantified by using a spectral photometer (JASCO Corporation, MODEL: PTL-3965, Tokyo, Japan).

### Statistical analysis

All data are expressed as the mean ± SE. Significant differences among each group were examined using a one-way of analysis of variance (ANOVA) followed by Bonferroni multiple comparison. A probability value of *P* < 0.05 was considered to indicate statistical significance.

## Results and Discussion

### Effect of HSA–Trx on body weight and survival of influenza-infected mice

**Figure 1:**
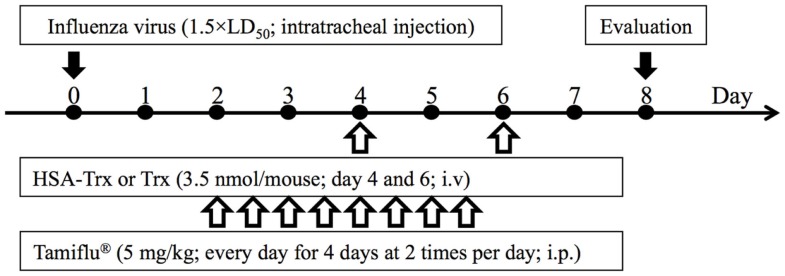
**Experimental protocol for the effective evaluation of HSA–Trx, Trx, or Tamiflu^®^ on influenza-induced ALI model mice**. Either HSA–Trx or Trx was administrated intravenously (1.75 nmol/mouse) at 4 and 6 days after virus infection. Tamiflu^®^ was administrated intraperitoneally (10 mg/kg/day, 2 times/day) for a total of 4 days, starting 2 days after the virus infection. The efficacy of therapeutic agents was evaluated with mice sacrificed at 8 days after virus infection.

Acute lung injury was induced in mice by a single intratracheal administration of influenza virus at a dose of 1.5 × LD_50_ (day 0). Since Yashiro et al. reported that the multiple administration of Trx (3.5 nmol/body) prevented influenza-induced ALI (15), we adopted a dose of HSA–Trx equivalent to one Trx treatment (3.5 nmol/body). In Figure S1 in Supplementary Material, the time-dependent variation following the virus infection shows that the infiltration of inflammatory cells, especially neutrophils in BALF, lung edema, and injury was induced from day 4 to 6 after the initial virus infection. In addition, as shown in Figure S1D in Supplementary Material, the virus load after influenza infection reached a peak at day 4, and then decreased. Therefore, HSA–Trx was injected at days 4 and 6 after the virus infection (Figure 1).

**Figure 2:**
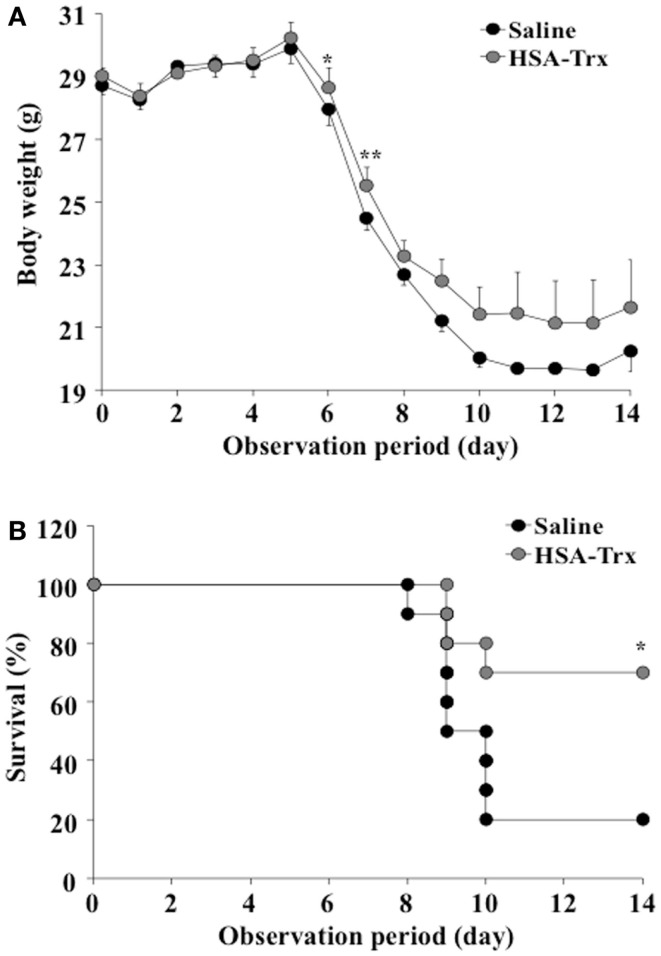
**Effect of HSA–Trx on the body weight (A) and survival (B) of influenza-infected mice**. HSA–Trx (3.5 nmol/mouse) was administrated intravenously at 4 and 6 days after the virus infection. **P < 0.01 or *P < 0.05 as compared with saline (*n* = 10).

As shown in Figure 2, the body weight of the saline treated group mice decreased from day 6 after the virus infection, with 80% of them dead. On the other hand, HSA–Trx administration significantly suppressed the diminished body weight and increased the survival rate in virus-infected mice. Finally, 70% of the HSA–Trx treated mice were still alive at 14 days after the virus infection.

### Relative inhibitory effect of HSA–Trx or Trx on influenza-infected mice

**Figure 3:**
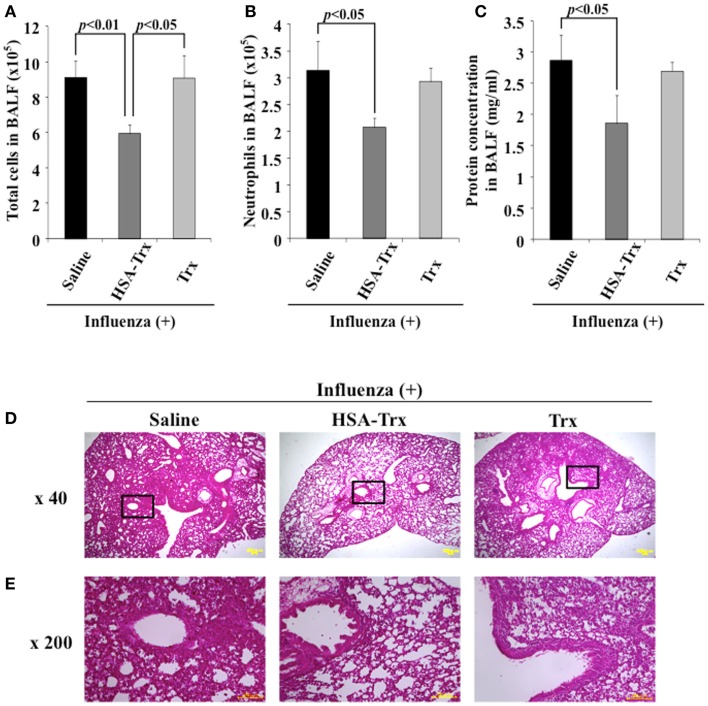
**Relative inhibitory effect of HSA–Trx or Trx on influenza-infected mice**. The same injected dose (3.5 nmol/mouse) of HSA–Trx or HSA was administered intravenously at 4 and 6 days after the virus infection. The numbers of **(A)** total cells and **(B)** neutrophils, or **(C)** protein concentration in BALF were determined at 8 days after virus infection. **(D)** Sections of pulmonary tissue were prepared at 8 days after virus infection, and subjected to histopathological examination (HE staining). Magnifications: ×40 in **(D)**; ×200 in **(E)**. Each value represents the mean ± SE (*n* = 5).

We compared HSA–Trx vs. Trx for their protective effect at the same administration schedule against influenza-induced ALI mice (Figure 1). The influenza infection caused a significant induction in the infiltration of inflammatory cells, especially neutrophils in the BALF of infected mice on day 8 (Supporting Figure in Supplementary Material). The number of total cells and neutrophils in the BALF remained significantly lower in HSA–Trx treated mice compared with saline or Trx treated mice (Figures 3A,B).

The extent of acute lung damage elicited by the influenza virus infection was assessed based on the increase in total protein in the BALF. We compared the levels of protein in the BALF from the saline, HSA–Trx, or Trx treated mice. As shown in Figure 3C, HSA–Trx significantly suppressed the elevation in the BALF protein that was induced by the influenza virus. The Trx treatment failed to result in a significant reduction in the virus-induced elevation of protein concentration in the BALF (Figure 3C).

Hematoxylin and eosin staining of lung sections indicated that the virus-inoculated mice presented diffuse edema and inflammatory infiltration in the alveoli and interstitium of the lung, hemorrhaging, and thickened airways on day 8 (Supporting Figure in Supplementary Material). However, in lung sections from the HSA–Trx administration group, the extent of damage was attenuated, compared with the saline treated group. Histopathological findings for lung sections from the Trx administration group were similar to that for the saline administration group (Figures 3D,E). This tendency was similar to the previous findings for OVA or BLM-induced lung injuries, in that HSA–Trx ameliorated these lung injuries in an administration schedule in which Trx failed to show any efficacy against these injuries (21, 22). Therefore, the suppressive effect of HSA–Trx may be due to the extended retention of the fusion protein in the blood, compared to HSA via FcRn.

### Effect of HSA–Trx on BALF IFN-γ and pulmonary iNOS expression

**Figure 4:**
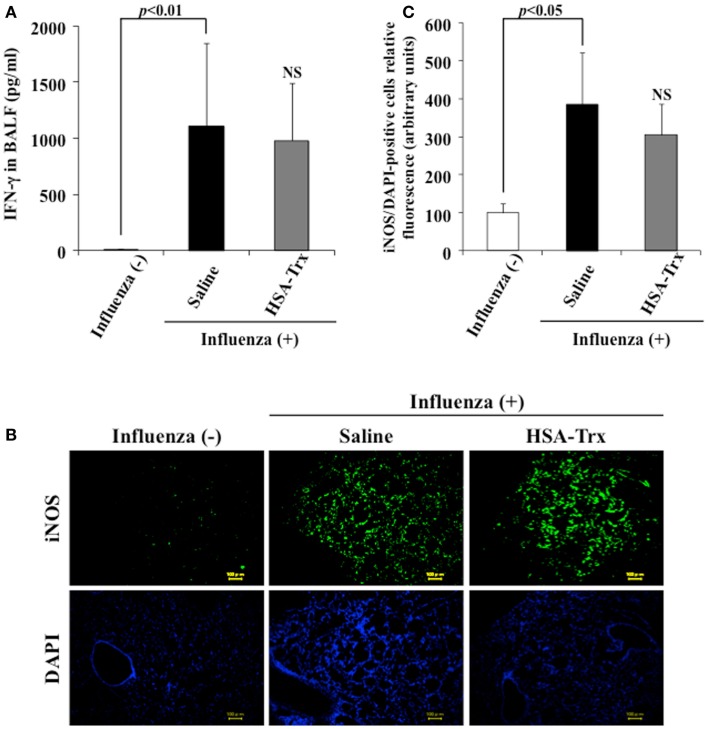
**Effect of HSA–Trx on BALF IFN-γ and pulmonary iNOS expression in influenza-infected mice**. **(A)** IFN-γ levels in BALF were determined, and **(B)** immunostaining of lung slice for iNOS/DAPI was performed at 8 days after the influenza infection. **(C)** Image analysis of the extent and intensity of iNOS/DAPI staining was performed. Each bar represents the mean ± SE [**(A)** *n* = 5, **(C)** *n* = 4]. NS; non-significant vs. saline.

A previous report indicated that a large amount of IFN-γ was induced in BALF obtained from influenza virus infections, and IFN-γ up-regulated the expression of iNOS in alveolar macrophages, bronchial, and alveolar epithelial cells (9, 23). To reveal the mechanism underlying the suppressive effect of HSA–Trx on influenza-induced ALI, the levels of IFN-γ in BALF or the expression of iNOS in lung tissue was confirmed by ELISA or immunostaining, respectively. As shown in Figure 4, the administration of HSA–Trx failed to reduce the increased levels of expressed IFN-γ and iNOS that were produced as the result of a viral infection.

### Effect of HSA–Trx on oxidative stress in lung tissue

Previous reported findings suggest that ROS released from activated leukocytes, especially alveolar macrophages and neutrophils, are associated with the development of influenza-induced ALI (6). In fact, the genetic knockout of NADPH-oxidase in which neutrophils are produced, resulted in the suppression of influenza-induced ALI (24). In addition, NO released by iNOS reacts with $O_{2}^{•-}$ derived from activated leukocytes to produce peroxynitrite (ONOO^−^) that has a strong injurious activity. ONOO^−^ can nitrate a wide range of biological targets, such as proteins and fatty acids to produce NO_2_-Tyr adducts (25).

**Figure 5:**
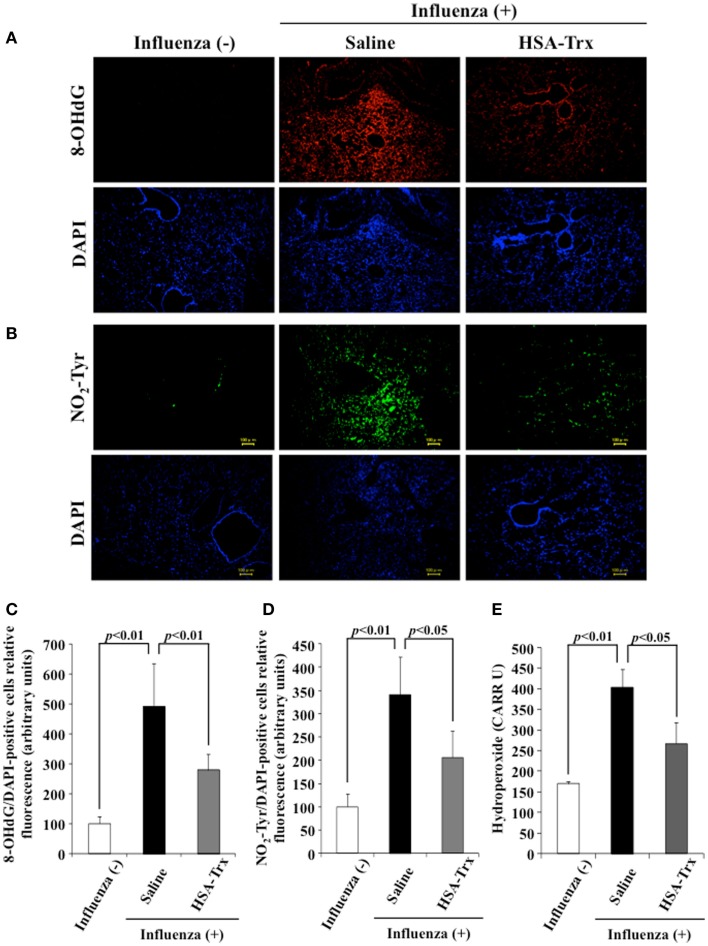
**Effect of HSA–Trx on pulmonary and plasma oxidative stress in influenza-infected mice**. Immunostaining of lung slice for **(A)** 8-OHdG/DAPI and **(B)** NO_2_-Tyr/DAPI was performed at 8 days after the influenza infection. Image analysis of the extent and intensity of **(C)** 8-OHdG/DAPI and **(D)** NO_2_-Tyr/DAPI staining was performed. **(E)** Plasma hydroperoxide level was determined at 8 days after influenza infection. Each bar represents the mean ± SE (*n* = 4).

To evaluate the effect of HSA–Trx on the oxidative stress induced by a virus infection, lung tissue was immunostained for 8-OHdG and NO_2_-Tyr, an oxidized product of nucleic acids and proteins, respectively, on day 8 after the influenza infection. Additionally, to determine the levels of hydroperoxide in plasma, dROMs tests were performed. As shown in Figure 5, the expression of 8-OHdG and NO_2_-Tyr in lung tissue and plasma hydroperoxides levels were increased in influenza-infected mice compared to normal mice, while HSA–Trx clearly suppressed the levels of these oxidative stress markers. Our previous report suggested that HSA–Trx has direct scavenging activities against $\left(O_{2}^{•-}\right)$ derived from activated neutrophils and that this effect is concentration dependent (22). Therefore, the inhibitory effect of oxidized products that accumulated was thought to involve the same mechanism as proposed in a previous report. Furthermore, Trx has anti-chemotaxis properties for neutrophils by inhibiting the activation of p38 mitogen-activated protein kinase, the downregulation of L-selectin (CD62L) and adhesion to endothelial cells (13). In fact, as shown in Figure 3B, HSA–Trx significantly lowered the number of neutrophils in the BALF of influenza-infected mice. Taken together, the mechanism underlying the improving effect of HSA–Trx on influenza-induced ALI could be not only attributed exclusively to an anti-oxidative effect but also anti-chemotaxis appears to be involved.

### Relative inhibitory effect of HSA–Trx or Tamiflu^®^ on influenza-infected mice

**Figure 6:**
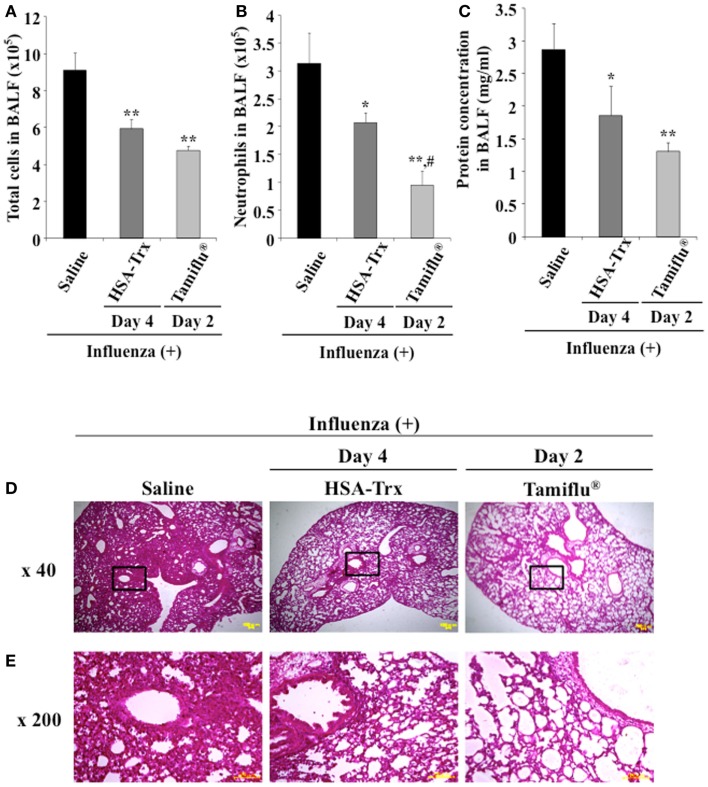
**Relative inhibitory effect of HSA–Trx or Tamiflu^®^ on influenza-infected mice**. HSA–Trx (3.5 nmol/mouse) was administered intravenously at 4 and 6 days after virus infection. Tamiflu^®^ (5 mg/kg) was administrated intraperitonealy every day at 2 times per day for a total of 4 days starting on 2 day after the influenza infection. The numbers of **(A)** total cells and **(B)** neutrophils, or **(C)** protein concentration in BALF were determined at 8 days after virus infection. **(D)** Sections of pulmonary tissue were prepared at 8 days after virus infection, and subjected to histopathological examination (HE staining). Magnifications: ×40 in **(D)**; ×200 in **(E)**. Each value represents the mean ± SE (*n* = 5). *P < 0.05 and **P < 0.01 as compared with saline. ^#^*P* < 0.05 as compared with HSA–Trx.

For future clinical applications, the inhibitory effect of HSA–Trx against influenza-induced ALI was examined. A previous study demonstrated that the intraperitoneal administration of Tamiflu^®^ (5 mg/kg) daily at two times per day showed a protective effect for influenza-induced ALI (26). Hence, the inhibitory effect of HSA–Trx (3.5 nmol/mouse, i.v.) at 4 and 6 days against influenza-induced ALI was compared with that of Tamiflu^®^ (5 mg/kg), which was administered every day at 2 times per day for 4 days from 2 day after the influenza infection (Figure 1). The evaluation items were the same as Figure 3. As shown in Figure 6, Tamiflu^®^ also significantly suppressed the derivation of inflammation cells and neutrophils infiltration, protein elevation in BALF and lung histopathological alteration caused by the influenza virus. The suppressive effect of Tamiflu^®^ treatment group was slightly stronger than that of HSA–Trx.

### Effect of HSA–Trx or Tamiflu^®^ on viral titers

**Figure 7:**
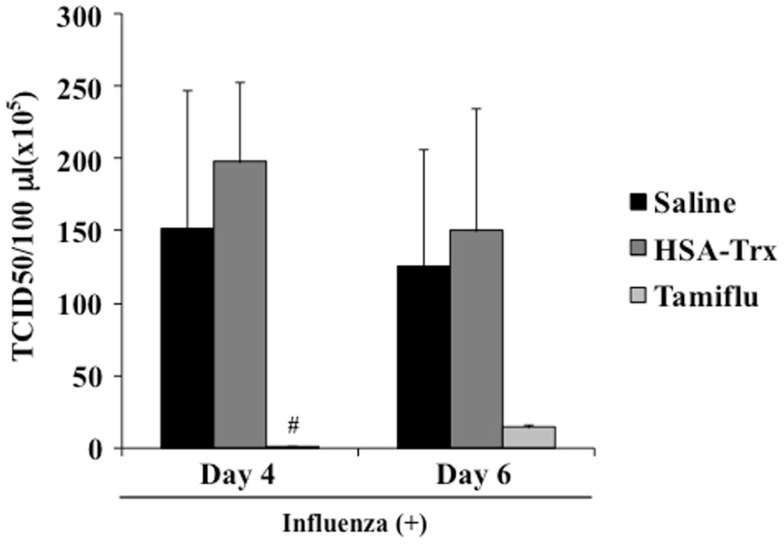
**Effect of HSA–Trx or Tamiflu^®^ on pulmonary virus replication in influenza-infected mice**. HSA–Trx (3.5 nmol/mouse) was administered intravenously at 4 and 6 days after the virus infection. Tamiflu^®^ (5 mg/kg) was administrated intraperitonealy every day at 2 times per day for a total of 4 days starting on 2 day after the influenza infection. The virus titles in lung tissue were determined at 4 and 6 days after the virus injection. Each value represents the mean ± SE (*n* = 5).

To validate that HSA–Trx treatment did not inhibit viral replication, we performed a plaque-forming assay in MDCK cells using the lung supernatant recovered from mice at day 4 or 6 after the virus infection. As shown in Figure 7, although the Tamiflu^®^ treatment markedly inhibited virus proliferation, no significant difference between the lung viral titers in the Saline and HSA–Trx administration mice was found. These results indicate that HSA–Trx, unlike a Tamiflu^®^, treatment ameliorates the acute lung damage induced by the virus infection without affecting viral propagation in the lungs of these mice.

### BALF distribution of FITC-labeled HSA–Trx in influenza-infected mice

**Figure 8:**
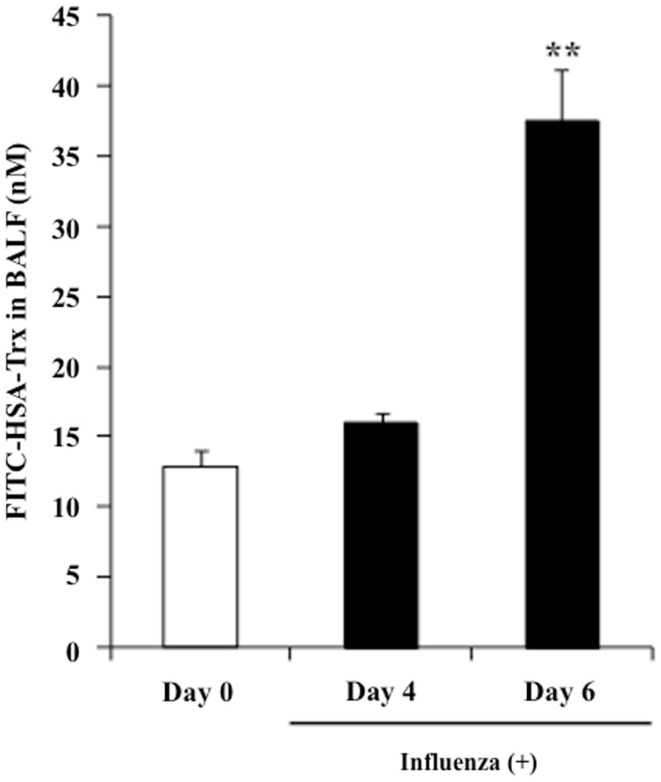
**BALF distribution of FITC-labeled HSA–Trx in influenza-infected mice**. BALF levels of FITC-labeled HSA–Trx after intravenous administration into the tail vein of normal (Day 0) or influenza-infected (Day 4 or 6) mice were determined using fluorescence spectroscopy. Each point represents the mean ± SE (*n* = 4). **P < 0.01 vs. Day 0.

The concentration of albumin in alveolar fluid is usually much lower than that in the blood, whereas the concentration can increase to 75–95% of the plasma level in the case of a lung injury due to vascular hyperpermeability caused by a marked elevation in the surfactant’s surface tension (27). In fact, the albumin concentration in BALF is used as a bio-marker of lung injuries in clinical settings. Thus, it would be predicted that the BALF distribution of exogenous HSA–Trx in influenza-infected mice would be higher than that in influenza-non-infected mice. Hence, FITC-labeled HSA–Trx was prepared, and the distribution to BALF at 2 h after its administration in control mice (Day 0) or model mice on day 4 (Day 4) or 6 (Day 6) after the virus infection was evaluated. As shown in Figure 8, the BALF distribution of FITC-labeled HSA–Trx at Day 6 was approximately 4 times higher than that at Day 0. This result suggests that the distribution of HSA–Trx to the site of the lesion, the interstitium in influenza-infected patients may be higher than that in healthy subjects.

## Conclusion

We evaluated the therapeutic effects of HSA–Trx, a long-lasting anti-oxidant and anti-inflammatory modulator that acts via the efficient receptor-mediated recycling pathway involving FcRn, on influenza-induced ALI model mice, and reached four major findings; (1) The post-administration of HSA–Trx suppressed influenza-induced ALI, (2) The mechanism by which HSA–Trx inhibited influenza-induced ALI can be attributed to a combination of anti-oxidative and anti-chemotaxis effects, (3) Unlike Tamiflu^®^ the HSA–Trx treatment ameliorated acute lung damage induced by the virus infection without affecting viral propagation in the lung of these mice, (4) The distribution of HSA–Trx to the lesion site, namely the interstitium in influenza-infected patients may be higher than that in healthy subjects. Thus, we conclude that HSA–Trx has considerable potential for use as a therapeutic agent for the treatment of various acute inflammatory disorders such as influenza-virus-induced pneumonia.

## Conflict of Interest Statement

The authors declare that the research was conducted in the absence of any commercial or financial relationships that could be construed as a potential conflict of interest.

## Supplementary Material

The Supplementary Material for this article can be found online at http://www.frontiersin.org/Journal/10.3389/fimmu.2014.00561/abstract
