# Supplementary material for: Therapeutic Impact of Human Serum Albumin–Thioredoxin Fusion Protein on Influenza Virus-Induced Lung Injury Mice
Source: Front Immunol. 2014 Nov 5;5:561. doi: 10.3389/fimmu.2014.00561 (PMC4220708; doi:10.3389/fimmu.2014.00561)
Supplement: Supplementary file 1 [file Data_Sheet_1.DOCX]

**Therapeutic impact of Human serum albumin-thioredoxin fusion protein on influenza virus-induced lung injury mice**

**Supporting Information**

**Supporting Figure. Evaluation of influenza-induced ALI model mice:** The numbers of (A) total cells and (B) neutrophils, (C) protein concentration in BALF, or (D) the virus titers in lung tissue were determined 0, 2, 4, 6 and 8 days after the virus infection. (E) Sections of pulmonary tissue were prepared 2, 4, 6 and 8 days after the virus infection, and subjected to histopathological examination (HE staining). Magnifications: x40 in upper panel; x200 in lower panel. Each value represents the mean±SE (n=3). ***P*<0.01 as compared with Day 0.
